# T Cell Responses to SARS-CoV-2 in Vaccinated Pregnant Women: A Comparative Study of Pre-Pregnancy and During-Pregnancy Infections

**DOI:** 10.3390/vaccines12111208

**Published:** 2024-10-24

**Authors:** Ching-Ju Shen, Shu-Yu Hu, Chung-Ping Hou, Ching-Fen Shen, Chao-Min Cheng

**Affiliations:** 1 Department of Obstetrics and Gynecology, Kaohsiung Medical University Hospital, Kaohsiung Medical University, Kaohsiung 807, Taiwan; chenmed.tw@yahoo.com.tw; 2 Institute of Biomedical Engineering, National Tsing Hua University, Hsinchu 300, Taiwan; chloe.natshuun@gmail.com; 3 Genmall Biotechnology Co., Ltd., Taipei 114, Taiwan; victor.hou@genmall.com.tw; 4 Department of Pediatrics, National Cheng Kung University Hospital, College of Medicine, National Cheng Kung University, Tainan 701, Taiwan

**Keywords:** T cell response, T-SPOT.COVID test, SARS-CoV-2, pregnancy, neutralizing antibody

## Abstract

The COVID-19 pandemic has posed unprecedented challenges to global public health, particularly for vulnerable populations like pregnant women. This study delves into the T cell immune responses in pregnant women with confirmed SARS-CoV-2 infection, all of whom received three doses of a COVID-19 vaccine. Using the ELISpot assay, we measured T cell responses against SARS-CoV-2 spike S1 and nucleocapsid peptides in two groups: those infected before and during pregnancy. Our results showed weak to moderate correlations between T cell responses and neutralizing antibody levels, with no statistically significant differences between the two groups. T cell reactivity appeared to decrease over time post-diagnosis, regardless of infection timing. Intriguingly, over half of the participants maintained detectable T cell memory responses beyond one year post-infection, suggesting the long-term persistence of cellular immunity. These insights contribute to the understanding of COVID-19 immunology in pregnant women, highlighting the importance of considering both humoral and cellular immune responses in this high-risk population.

## 1. Introduction

The COVID-19 pandemic has posed unprecedented challenges to global public health, particularly for high-risk groups such as pregnant women. While vaccination has proven effective in preventing COVID-19, more research is needed on immune responses in pregnant women following SARS-CoV-2 infection. T cells are crucial in combating viral infections, especially in generating long-term immune memory [1]. Multiple studies have shown that robust SARS-CoV-2-specific T cell responses are associated with reduced COVID-19 severity [2]. T cell-mediated immunity can protect the host, even under insufficient humoral immune responses [3]. Compared to neutralizing antibodies, SARS-CoV-2-specific memory T cells demonstrate longer persistence. A study revealed that SARS-CoV-2-specific CD4+ and CD8+ T cell responses in COVID-19-convalescent individuals can last up to 10 months, regardless of disease severity [4]. Furthermore, T cell responses to SARS-CoV-2 variants are less affected due to the distribution of T cell epitopes across multiple structural and non-structural viral proteins.

Due to its high sensitivity and specificity, the enzyme-linked immunospot (ELISpot) technique has emerged as a gold standard for assessing T cell function. The T-SPOT.COVID test, which employs the ELISpot methodology, measures T cell responses to SARS-CoV-2 spike S1 and nucleocapsid peptides. Through ELISpot, we can accurately quantify specific T cell numbers and their cytokine secretion, providing insights into the immune response of pregnant women to SARS-CoV-2 infection.

Pregnancy presents a unique immunological state, requiring a delicate balance between fetal tolerance and maternal protection against infections. Understanding T cell responses in pregnant women who are infected with SARS-CoV-2 is crucial for developing targeted vaccination strategies and improving maternal and fetal outcomes. This study has investigated T cell responses in pregnant women with confirmed SARS-CoV-2 infection, comparing those who were infected before pregnancy with those who were infected during pregnancy, all of whom have received three doses of the COVID-19 vaccine. By examining these groups, we have aimed to elucidate the dynamics of cellular immunity in pregnant women with previous SARS-CoV-2 infection and assess the potential impact of pregnancy on vaccine-induced T cell responses. The findings would contribute to our understanding of COVID-19 immunology in this high-risk group, potentially informing vaccination strategies and enhancing our knowledge of immune responses during pregnancy in the context of prior infection and vaccination.

## 2. Materials and Methods

### 2.1. Study Design and Patients

The institutional review board of Kaohsiung Medical University Hospital approved this prospective study (IRB number: KMUHIRB-SV(II)-20210087). Using a convenience sampling approach, we collected samples from 24 pregnant women who delivered at Kaohsiung Medical University Hospital between February to July 2023. The inclusion criteria were (1) completion of three doses of monovalent SARS-CoV-2 vaccine prior to delivery, (2) confirmed SARS-CoV-2 infection either before or during pregnancy, (3) no history of chronic diseases, (4) absence of prenatal and intrapartum complications, and (5) willingness to participate in the study. These criteria ensured that our sample represented a group of generally healthy pregnant women with both vaccination and infection history. The convenience sampling method was chosen due to the specific nature of our target population and the constraints of the hospital setting. We used the T-SPOT.COVID test, Oxford Immunotec, to assess the extent of cellular immune responses and used ACROBiosystems’ anti-SARS-CoV-2 (BA.4. and BA.5) Neutralizing Antibody Titer Serologic Assay Kit (Spike RBD) to detect the neutralizing antibody inhibition rate among the 24 samples. Combining the above experimental methods, we explored the effect of SARS-CoV-2 on T cell responses through humoral immunity and cellular immunity.

### 2.2. Study Variables and Data Acquisition

The following data were obtained from the medical records: the mother’s age, weight, height, race, estimated date of confinement, parity, medical and obstetric history, interval of COVID-19 vaccination, date of pertussis immunization, date of influenza immunization, newborn’s sex, and newborn’s body weight. Maternal and cord blood-neutralizing antibody levels were determined as dependent variables.

### 2.3. T-SPOT.COVID Test

Regarding the T-SPOT.COVID test, we used a USB microscope with a mobile phone camera program to take photos. The number of dark points can be calculated based on the photo to determine whether a patient sample was ‘Reactive’ or ‘Non-reactive’. A normal result would be expected to have few or no spots in the negative control and ≥20 spots in the positive control. The neutralizing antibody inhibition rate (percentage) was calculated using the Neutralizing Antibody Titer Serologic Assay Kit, with the O.D. value being based on the following formula:

$$Percentage\;inhibition=\left(1-\frac{{O.D.}_{450\mathrm{nm}}-{O.D.}_{630\mathrm{nm}}\;of\;sample}{{O.D.}_{450\mathrm{nm}}-{O.D.}_{630\mathrm{nm}}\;of\;negative\;control}\right)\times 100\%$$

The T-SPOT.COVID experimental procedure began by drawing 3.5 mL of human whole blood, which must be processed within 52 h. The blood, buffer, and magnetic beads were mixed in a seven-row tube and left to stand for 20 min. Peripheral blood cells were isolated using a magnetic rod for 45 min after adding the cell culture media RPMI and AIM-V. Each well received 100 μL of cell suspension, ensuring 2.5 × 10^5^ cells per well, using a cell counter and AIM-V for dilution. Four data points were collected per sample: negative control, positive control, spike protein, and nucleocapsid protein. Each received 100 μL of sample and corresponding working solution, followed by a 20 h incubation at 37 °C with 5% CO_2_. Wells were then washed with 200 μL of D-PBS three times, followed by adding 50 μL conjugate solution and a one-hour incubation at 2–8 °C. Steps were repeated, and then, 50 μL of substrate solution was added and incubated for 7 min at room temperature. The wells were washed with deionized water and dried at 37 °C for 4 h, after which the results were recorded.

### 2.4. Statistical Analysis

Statistical analysis was performed using SPSS version 25.0 (IBM Corp., Armonk, NY, USA). We used X-Y plots and Pearson correlation coefficient analysis to analyze correlations between different experimental data from the same pregnant woman. We chose Pearson’s correlation based on the assumption of linear relationships between our continuous variables and the approximately normal distribution of our data. A two-tailed test was conducted for each correlation, with statistical significance set at *p* < 0.05. The correlations analyzed included the following: 1. T cell immune response and neutralizing antibody inhibition rate in the blood of confirmed pregnant women; 2. T cell immune response and the number of weeks since diagnosis. For each correlation, we calculated the Pearson correlation coefficient (r) and the corresponding *p* value. The *p* value indicates the probability of observing the calculated correlation coefficient by chance, with lower *p* values suggesting stronger evidence against the null hypothesis of no correlation. We used widely accepted criteria to interpret the strength of correlations based on the absolute value of the correlation coefficient (r). Correlations were classified as weak (r < 0.3), moderate (0.3 ≤ r < 0.7), or strong (r ≥ 0.7). These generally recognized standards were applied consistently throughout our analysis to classify the strength of the observed correlations.

## 3. Results

Table 1 presents the demographic and clinical characteristics of the study participants. The cohort comprised 24 pregnant women with laboratory-confirmed SARS-CoV-2 infection, divided into two groups: those diagnosed before pregnancy (N = 13) and those diagnosed during pregnancy (N = 11). Statistical analysis revealed no significant differences between these groups regarding age, parity, BMI, or gestational age at delivery. All participants received monovalent mRNA COVID-19 vaccines for their third dose. Notably, all cases who were diagnosed with COVID-19 before pregnancy had completed their three-dose vaccination series before conception. Of the 11 cases diagnosed during pregnancy, 10 had completed their three-dose vaccination before diagnosis, while 1 received the third dose 12 weeks after diagnosis.

**Figure 1:**
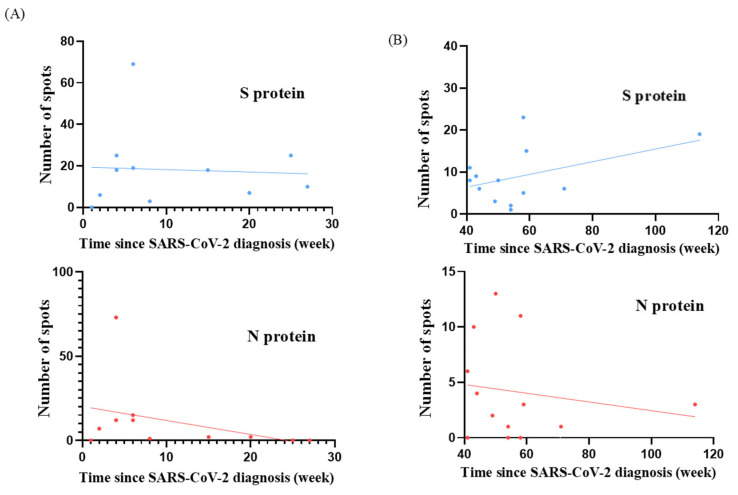
Scatter plots represent the relationship between T cell responses and (**A**) patients diagnosed during pregnancy and (**B**) before pregnancy. Data are shown for S protein and N protein responses in two groups: women diagnosed with SARS-CoV-2 during pregnancy (N = 11) and before (N = 13). The x-axis represents weeks since diagnosis, while the y-axis shows the number of reactive T cells (spots) for S and N proteins. Each point represents an individual participant. Table 2 provides the correlation coefficients (r) and *p* values.

**Figure 2:**
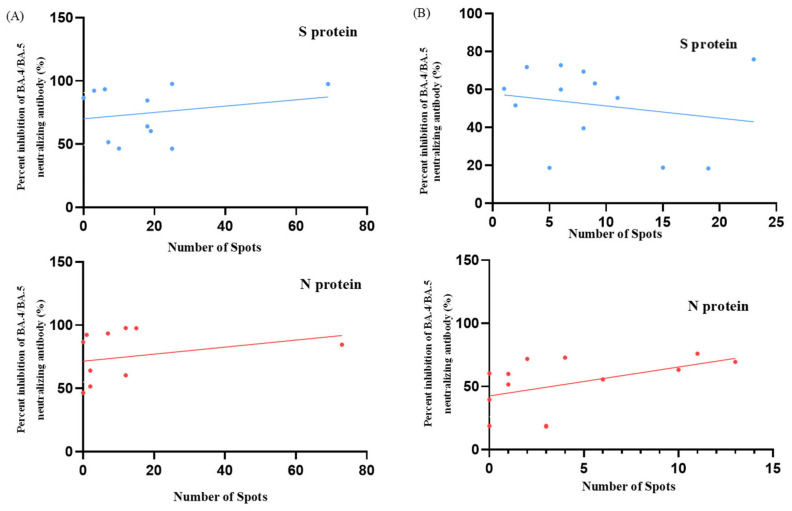
Scatter plots illustrating the correlation between BA.4/BA.5 neutralizing antibody inhibition rates and T cell responses for two groups: (**A**) women diagnosed with SARS-CoV-2 before pregnancy (N = 13) and (**B**) those diagnosed during pregnancy (N = 11). The plots display data for both S protein and N protein responses. The x-axis denotes the number of reactive T cells (spots) for S and N proteins, while the y-axis indicates the percentage of the BA.4/BA.5 neutralizing antibody inhibition rate in maternal blood. Individual data points represent single participants from each group.

T cell responses were evaluated in terms of the time since SARS-CoV-2 diagnosis and BA.4/BA.5 neutralizing antibody inhibition rates for both study groups. The scatter plots depict the distribution of individual participant data for S and N protein responses and are shown in Figure 1 and Figure 2. We analyzed the temporal relationship between T cell responses and time since SARS-CoV-2 diagnosis and the correlation between T cell responses and neutralizing antibody inhibition rates (Table 2 and Table 3). In the cohort diagnosed before pregnancy, we observed moderate correlations for S protein responses with time since diagnosis (r = 0.44; *p* = 0.13) and with neutralizing antibody inhibition rates (r = 0.20; *p* = 0.52). For N protein responses, we observed a trend towards weak correlations with time since diagnosis (r = 0.17; *p* = 0.58) and moderate correlations with neutralizing antibody inhibition rates (r = 0.48; *p* = 0.10). Conversely, in the group who were diagnosed during pregnancy, we found a negligible correlation between S protein responses and time since diagnosis (r = 0.06; *p* = 0.86) but a weak correlation with neutralizing antibody inhibition rates (r = 0.23; *p* = 0.50). For N protein responses, our analysis suggested a possible relationship with time since diagnosis (r = 0.37; *p* = 0.26), which could be described as moderate. This indicated a potential association with neutralizing antibody inhibition rates (r = 0.28; *p* = 0.40) that could be characterized as weak. However, it is essential to note that neither of these correlations reached statistical significance (all *p* > 0.05). These findings suggest that T cell responses, neutralizing antibody levels, and time since diagnosis may not be strongly interrelated in our cohort. The absence of solid and significant correlations between these aspects of the immune response highlights the complex nature of immunity to SARS-CoV-2 in pregnant women, regardless of whether infection occurred before or during pregnancy.

**Figure 3:**
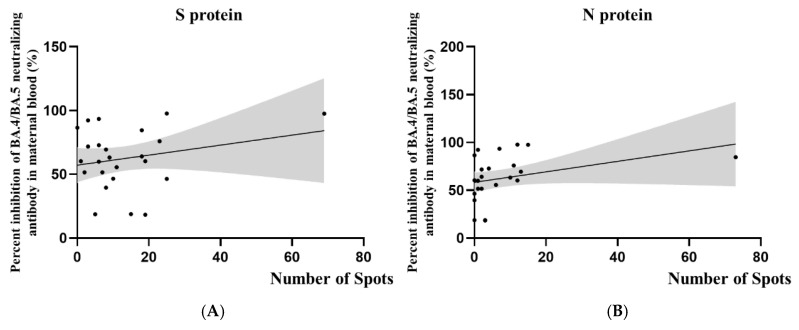
Correlation between SARS-CoV-2-specific T cell responses and BA.4/BA.5 neutralizing antibody inhibition rates. (**A**) Spike protein-specific and (**B**) nucleocapsid protein-specific T cell responses. X-axis: number of reactive T cells; Y-axis: percentage of BA.4/BA.5 neutralizing antibody inhibition rate.

We further analyzed the T cell responses in 24 women who had confirmed SARS-CoV-2 infection, including those who were diagnosed before and during pregnancy. Figure 3 illustrates the relationship between SARS-CoV-2-specific T cell responses and BA.4/BA.5 neutralizing antibody inhibition rates in this combined cohort. The scatter plots suggest a trend where higher neutralizing antibody inhibition rates correspond with more active cellular immune responses. However, it is crucial to note that this correlation did not reach statistical significance. As shown in Table 4, the correlation coefficients for S protein (r = 0.23; *p* = 0.27) and N protein (r = 0.34; *p* = 0.10) responses were weak to moderate, with *p* values above the conventional threshold for statistical significance.

**Figure 4:**
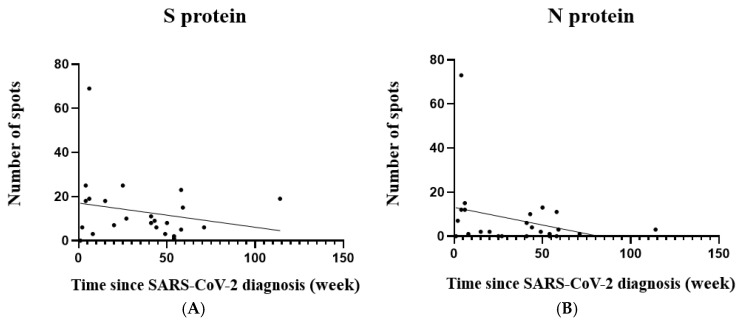
Relationship between SARS-CoV-2-specific T cell responses and time since diagnosis. (**A**) Spike protein-specific and (**B**) nucleocapsid protein-specific T cell responses. X-axis: number of reactive T cells; Y-axis: weeks since SARS-CoV-2 diagnosis.

Figure 4 depicts the association between T cell responses and the time since SARS-CoV-2 diagnosis for all 24 participants. The scatter plots for both S protein and N protein responses show a slight negative trend, indicating a potential decrease in T cell reactivity post-diagnosis, regardless of whether infection occurred before or during pregnancy. However, it is important to note that this trend did not reach statistical significance. This observation is supported by the negative correlation coefficients presented in Table 5 (S protein: r = −0.22 and *p* = 0.31; N protein: r = −0.30 and *p* = 0.15). However, it is essential to emphasize that these correlations are weak and did not reach statistical significance. While not definitive, these findings indicate a need for further investigation with larger sample sizes to better understand the T cell response dynamics in pregnant women with prior SARS-CoV-2 infection.

In our study data, among the 12 pregnant women who were diagnosed during pregnancy, only 2 had negative T cell response test results, yielding a positive rate of 83.3%. In contrast, among the 12 women who were diagnosed before pregnancy, 5 had negative test results, resulting in a positive rate of 58.3%. We observed that the probability of detecting positive T cell immunity decreased as the time since diagnosis increased. However, it is noteworthy that there was over a 50% chance of retaining T cell immune memory even after more than a year post-diagnosis. The most potent T cell responses, characterized by a total T cell count exceeding 50, were found to be all cases where 4–6 weeks had elapsed between diagnosis and blood sampling. This observation suggests a peak in T cell response during this time frame. Notably, there is a delay in the measurable T cell responses; no detectable response was observed in cases where blood samples were taken within one week of diagnosis. This pattern indicates a temporal progression in the development of T cell immunity, with responses becoming more robust several weeks after the initial infection.

## 4. Discussion

T cell responses exhibit unique regulatory patterns during pregnancy to balance the immune tolerance towards the fetus and defend against pathogens [5,6,7,8]. Certain viral infections may alter T cells’ functions during pregnancy, affecting their ability to clear pathogens and maintain immune tolerance towards the fetus [9,10,11,12,13,14]. Pregnancy may influence the formation and maintenance of T cell memory responses, which has important implications for vaccination strategies and defense against reinfection. Our research has aimed to elucidate the specific T cell responses to SARS-CoV-2 in pregnant women, a population with uniquely adapted immune systems. We sought to investigate how pregnancy-induced immunological changes influence the response to viral infection, comparing women who were infected before and during pregnancy, all of whom received COVID-19 vaccines. Comparing immune responses across vulnerable populations can provide valuable insights into SARS-CoV-2’s pathogenesis. Recent research by Oishi et al. (2024) on pediatric populations revealed significant differences in their cellular immune responses to SARS-CoV-2 compared to adults, with children experiencing mild cases and potentially not developing substantial cellular immunity [15]. This presents an intriguing contrast to our observations in pregnant women, who exhibit unique patterns of cellular immunity due to pregnancy-induced immune modulation.

While previous studies have examined immune responses to SARS-CoV-2 in pregnant women, our research offers several unique contributions to the field. Unlike Collier et al., who focused solely on vaccine-induced immunity, our study provides a comprehensive view of natural infection and vaccination effects [16]. We precisely documented the infection timing, distinguishing between pre-pregnancy and during-pregnancy infections, a crucial factor that has not been addressed in previous works. This allows for a nuanced analysis of how the infection timing influences immune responses. In contrast to Hsieh et al.’s smaller cohort, our larger sample size of 24 subjects, all of whom received three vaccine doses, enables more robust statistical analyses and potentially more generalizable findings [17]. Furthermore, while Chambers et al. primarily examined post-vaccination T cell responses, our study investigates the interplay between known infection time points and vaccination status [18]. This approach offers a longer-term view of immune responses, from infection through to delivery, providing valuable insights into the evolution of immunity throughout pregnancy. By directly correlating the infection timing, vaccination status, and immune responses, our study offers more targeted clinical guidance for managing pregnant women with confirmed SARS-CoV-2 infections. These distinctive features of our research address critical gaps in understanding the immune response to SARS-CoV-2 in pregnant women, particularly in natural infection and vaccination.

We employed the T-SPOT.COVID assay, a highly sensitive and specific enzyme-linked immunospot (ELISpot) technique, to investigate these T cell responses [19,20]. The ELISpot assay, which has gained considerable traction recently, is versatile and robust across various immunological disciplines. It demonstrates its growing importance in cancer immunotherapy, infectious disease vaccine development, and autoimmune disease research [21]. The unique ability of the ELISpot assay to detect and enumerate antigen-specific T cells at the single-cell level provides a distinct advantage in assessing the nuanced immune responses in our cohort of pregnant women with COVID-19. Furthermore, the potential for clinical application of ELISpot-based diagnostics adds a translational dimension to our findings, enhancing their relevance in the broader context of COVID-19 management in high-risk populations.

Our study has found correlation coefficients of 0.2342 and 0.3423 between neutralizing antibodies and spike and nucleocapsid proteins, respectively. This low positive correlation reflects the individual variability in cellular immune responses. Previous studies have highlighted the distinct dynamics of B and T cell responses in COVID-19 immunity. GeurtsvanKessel et al. demonstrated that B cell responses produce robust antibody levels following vaccination, which decline and show reduced neutralization against variants like Omicron. In contrast, T cell responses remain stable and resilient to viral mutations, emphasizing their crucial role in mitigating severe COVID-19 when antibody responses wane [22]. Complementing these findings, Oja et al. (2020) observed divergent B and T cell responses following natural SARS-CoV-2 infection. While mild cases exhibited coordinated humoral and cellular responses, critically ill patients displayed a striking imbalance characterized by elevated antibody titers but diminished SARS-CoV-2-specific T cell responses [23]. This dysregulation in severe cases, manifested as impaired T cell functionality and altered tissue distribution, underscores the complex interplay between humoral and cellular immunity in COVID-19’s pathogenesis. These observations provide a crucial framework for our current investigation into the dynamics and functional implications of B and T cell responses in COVID-19 patients, particularly in the context of long-term immunity.

Previous studies on SARS-CoV-1 demonstrated the long-term persistence of virus-specific memory T cells [24,25,26]. Recent research on SARS-CoV-2 reveals similar patterns, suggesting a comparable durability of T cell responses [27,28,29,30]. Relying solely on seroprevalence may underestimate the true extent of adaptive immunity to SARS-CoV-2, as T cell responses can persist even without detectable antibodies [31]. While individuals with prior vaccination or infection generally maintain some level of neutralizing activity against most variants, the sensitivity of these antibodies varies across different viral strains [29,30,31]. T cell-mediated immune responses demonstrate stronger cross-reactivity across variants than antibody responses [32,33,34]. This enhanced T cell reactivity may be crucial in protecting against severe disease, even when the antibody effectiveness wanes.

Our findings align with the growing evidence suggesting that T cell responses play a crucial role in SARS-CoV-2 immunity. While we observed varying levels of neutralizing antibodies in our cohorts of pregnant women who were infected before and during pregnancy, the T cell responses did not always correlate strongly with antibody levels. This discordance underscores the importance of assessing both humoral and cellular immunity, as relying solely on seroprevalence may underestimate the true extent of adaptive immunity to SARS-CoV-2. The moderate to weak correlations observed between T cell responses and neutralizing antibody levels, regardless of infection timing, suggest that T cell immunity may persist even when antibody levels are low or undetectable. This observation provides valuable insights into the immune dynamics of SARS-CoV-2 in pregnant women, highlighting the importance of studying both humoral and cellular immunity in this unique population. Furthermore, our observation of T cell responses to both S and N proteins suggests a broad cellular immune response, which may contribute to the cross-reactivity against different SARS-CoV-2 variants reported in previous studies. Notably, we identified one case in our study where a participant with a negative T cell response experienced two SARS-CoV-2 infections during pregnancy, separated by an 18-week interval. This case highlights the potential importance of T cell immunity in preventing reinfection. It suggests that the absence of a robust T cell response may leave individuals more susceptible to subsequent infections, even within a relatively short timeframe. This observation aligns with the concept that T cell-mediated immunity may provide long-lasting protection against severe disease and reinfection, even as the antibody effectiveness potentially wanes. Although our study did not directly assess variant-specific responses, the persistence of T cell responses that we observed in most cases, particularly in women who were infected before pregnancy, supports the idea that T cell-mediated immunity may offer broader protection against SARS-CoV-2 and its variants. This case of reinfection in the absence of a T cell response further emphasizes the need for comprehensive immune profiling in pregnant women, considering both antibody and T cell responses, to better understand their protection against SARS-CoV-2 and its variants and to identify those who might be at a higher risk of reinfection.

Our study has several limitations. A primary limitation of this study is the relatively small sample size (n = 24). Nevertheless, our post hoc power analysis revealed a statistical power of 0.86 at α = 0.05, exceeding the commonly accepted threshold of 0.80. This indicates that despite the small sample, our study had sufficient power to detect expected effects. However, we acknowledge that the smaller sample size may limit the generalizability of our findings and could affect the ability to detect more subtle differences between groups. Future studies should consider larger samples to validate these findings further and extend them. Second, our study provides a cross-sectional view of immune responses at a single time point. Longitudinal follow-up could offer more insights into the evolution of T cell responses over time in pregnant women, particularly concerning the timing of infection and vaccination. Third, while we focused on T cell responses and neutralizing antibodies, our study did not explore other aspects of immunity, such as B cell memory or innate immune responses. A more comprehensive assessment of the immune landscape could provide a fuller picture of SARS-CoV-2 immunity in pregnant women. Fourth, while our study design compared two groups of vaccinated pregnant women—those who were infected with SARS-CoV-2 before pregnancy and those who were infected during pregnancy—it has both strengths and limitations. This approach allows us to explore how the timing of infection influences immune responses and to study the effect of time on the persistence of SARS-CoV-2 immune responses. However, the lack of a non-pregnant control group limits our ability to attribute observed immunological features specifically to pregnancy. Despite this limitation, our findings reflect real-world scenarios faced by vaccinated pregnant women and may have potential clinical implications for guiding vaccination strategies and infection management in this population. To address these limitations and further elucidate the impact of pregnancy on SARS-CoV-2 immune responses, future studies should consider including a non-pregnant control group and conducting longitudinal investigations. Lastly, while we inferred that Omicron variants predominantly caused infections during our study period, based on epidemiological data from the Taiwan CDC in the region where our study participants were located, we did not perform direct genetic sequencing of the virus strains. This limitation prevents us from determining specific subtypes and their potential impacts on immune responses.

## 5. Conclusions

This study highlights the crucial role of T cell immunity in SARS-CoV-2 infection during pregnancy, a period characterized by distinct immunological challenges. Our research uniquely examines SARS-CoV-2 immune responses in pregnant women by investigating natural and vaccination effects while precisely documenting the infection timing. By distinguishing between pre-pregnancy and during-pregnancy infections, this novel approach offers unprecedented insights into the development of immunity throughout pregnancy, addressing significant gaps in our current knowledge.

This study underscores the critical role of T cell immunity in the context of SARS-CoV-2 infection during pregnancy, a period marked by unique immunological challenges. The weak to moderate correlations between T cell responses and neutralizing antibody levels underscore the complex interplay between humoral and cellular immunity in this unique population. The persistence of T cell responses in most cases, even after more than a year post-diagnosis, suggests a potential role for cellular immunity in long-term protection against SARS-CoV-2. However, the gradual decline in T cell reactivity over time underscores the need to monitor immune responses in this high-risk group. Given that the correlation trends we observed did not reach statistical significance, these results should be considered preliminary observations requiring larger-scale studies for further validation. Our results highlight the need for tailored vaccination strategies that account for pregnancy’s unique immunological state. Although not statistically significant, the observed trends in immune responses between women who were infected before and during pregnancy suggest the potential importance of considering the infection timing when developing protective measures. While our study contributes to understanding SARS-CoV-2 immunity in pregnant women, it also underscores the need for more extensive, longitudinal studies to further elucidate the long-term implications of these immune responses on maternal and fetal outcomes. Future research should focus on optimizing vaccination protocols for pregnant women and investigating the impact of emerging SARS-CoV-2 variants on cellular immunity in this high-risk population. In conclusion, our findings provide a foundation for refining approaches to COVID-19 prevention and management in pregnant women, emphasizing the critical role of both humoral and cellular immunity in protecting this vulnerable group.

## Figures and Tables

**Table 1:** Summary of the demographic and clinical characteristics of the study participants. This study cohort comprised 24 pregnant women: 13 who were infected with SARS-CoV-2 before pregnancy and 11 who were infected during pregnancy.

Variable	Diagnosed Before Pregnancy ^a^	Diagnosed During Pregnancy ^b^
Age	31.08 * (±5.11 **)	32.82 * (±5.51 **)
Parity	1.38 * (±0.51 **)	1.00 * (±0.00 **)
BMI	27.56 * (±3.99 **)	26.36 * (±3.68 **)
Weeks of gestation at delivery	38.31 * (±0.63 **)	38.27 * (±0.90 **)
Interval between the infection of COVID-19 and the collection of blood sample (day of delivery) (weeks)	56.62 * (±19.27 **)	10.73 * (±9.41 **)
Interval between the first dose of COVID-19 vaccination and the collection of blood sample (day of delivery) (weeks)	101.23 * (±9.77 **)	73.73 * (±22.31 **)
Interval between the second dose of COVID-19 vaccination and the collection of blood sample (day of delivery) (weeks)	88.62 * (4.29 **)	65.00 * (±21.24 **)
Interval between the third dose of COVID-19 vaccination and the collection of blood sample (day of delivery) (weeks)	68.77 * (±10.35 **)	50.09 * (±20.43 **)

BMI: body mass index; * Mean; ** Standard Deviation(± SD); ^a^ Case number = 13; ^b^ Case number = 11.

**Table 2:** Correlation coefficients and *p* values of Figure 1.

Variant	T Cell Response	r *	*p* Value	N **
Diagnosed during pregnancy (Time since SARS-CoV-2 diagnosis)	S protein	0.06	0.86	11
	N protein	0.37	0.26	11
Diagnosed before pregnancy (Time since SARS-CoV-2 diagnosis)	S protein	0.44	0.13	13
	N protein	0.17	0.58	13

* r: Pearson’s correlation coefficient; ** N: case number.

**Table 3:** Correlation between T cell response and neutralizing antibody inhibition rate.

Variant	T Cell Response	r **	*p* Value	N ***
% of inhibition rate of Nabs * (during pregnancy)	S protein	0.23	0.50	11
	N protein	0.28	0.40	11
% of inhibition rate of Nabs * (before pregnancy)	S protein	0.20	0.52	13
	N protein	0.48	0.10	13

* Nabs: neutralizing antibodies; ** r: Pearson’s correlation coefficient; *** N: case number.

**Table 4:** Correlation between T cell response and neutralizing antibody inhibition rate.

Variant	T Cell Response	r **	*p* Value	N ***
% of inhibition rate of Nabs * (during pregnancy)	S protein	0.23	0.27	24
	N protein	0.34	0.10	24

* Nabs: neutralizing antibodies; ** r: Pearson’s correlation coefficient; *** N: case number.

**Table 5:** Correlation between T cell response and time since SARS-CoV-2 diagnosis.

Variant	T Cell Response	r *	*p* Value	N **
Time since SARS-CoV-2 diagnosis	S protein	−0.22	0.31	24
	N protein	−0.30	0.15	24

* r: Pearson’s correlation coefficient; ** N: case number.

## Data Availability

The original contributions presented in the study are included in the article; further inquiries can be directed to the corresponding authors.
